# An intelligent approach for analyzing the impacts of the COVID-19 pandemic on marketing mix elements (7Ps) of the on-demand grocery delivery service

**DOI:** 10.1007/s40747-021-00358-1

**Published:** 2021-04-09

**Authors:** Burak Can Altay, Abdullah Okumuş, Burcu Adıgüzel Mercangöz

**Affiliations:** 1 Department of Transportation, Istanbul University, Istanbul, Turkey; 2 Department of Marketing, Istanbul University, Istanbul, Turkey; 3 Department of Logistics, Istanbul University, Istanbul, Turkey

**Keywords:** COVID-19, Fuzzy AHP, Online grocery shopping, Mobile technology, On-demand grocery delivery, Marketing mix

## Abstract

Due to the impact of the COVID-19 pandemic, on-demand grocery delivery service that combines mobile technology and city logistics has gained tremendous popularity among grocery shoppers as a substitute to self-service grocery shopping in the store. This paper proposes an intelligent comparative approach where fuzzy logic and the analytical hierarchy process (AHP) method are combined to determine the importance weights of the criteria for marketing mix elements (7Ps) of the on-demand grocery delivery service for the period before COVID-19 and during COVID-19. In addition to its comprehensive theoretical insight, this paper provides a practical contribution to decision makers who create a marketing mix for the on-demand grocery delivery service and other similar online grocery businesses in terms of efficient allocation of resources to the development of marketing mix elements. The study’s findings can also provide clues for the decision makers in times of similar pandemics and crises that are likely to be seen in the future.

## Introduction

The coronavirus pandemic (COVID-19) has significantly impacted daily life. Many Turkish consumers, along with consumers in other countries, have changed their shopping habits to save their lives and prevent the further spread of COVID-19. According to a report on retail commerce sales, retail e-commerce in Turkey will develop with a compound annual growth rate (CAGR) of 20.2 between 2020 and 2024, while global CAGR during the same period will be 8.1% [64].

The pandemic is forcing more consumers to prefer e-commerce to meet their grocery needs, and it is expected that this rising trend will continue beyond the pandemic. According to a report published in July 2020, about one-quarter of the Turkish population do their grocery shopping online [65].

On-demand grocery delivery companies serve their customers by providing instant delivery of groceries and other goods through an app that enables users to order and pay for products with secure payment methods from the comfort of their homes. These companies need to determine an effective marketing mix strategy to gain superiority over their competitors in terms of sales share [45] and other critical targets.

This study aims to determine criteria for marketing mix elements of on-demand grocery delivery service and compute the importance weights of the criteria for marketing mix elements of on-demand grocery delivery service for the period before COVID-19 and during COVID-19. It is also aimed to present how the importance weights of these criteria have changed between these two periods.

Despite the increasing popularity of online grocery shopping, studies on determining the importance weights of the criteria for marketing mix elements of online grocery shopping are quite limited. Most studies in the literature are on the adoption of online grocery shopping [26, 59], customer satisfaction [3, 63], consumer decision-making [5, 29, 52], service quality [47], and success criteria [14].

Considering the lack of literature as well as COVID-19 being a new phenomenon with its effects on the grocery sector only recently observed, this paper is expected to remedy this gap by evaluating critical factors comparatively before COVID-19 and during COVID-19.

Therefore, the study is valuable in showing which criteria have gained importance due to the COVID-19 pandemic. The remainder of the study is structured in the following manner. The next section describes the theoretical background of the study and compasses the related literature. The third section addresses the research methodology, including a detailed explanation of Fuzzy Theory and Buckley Extension-Based Fuzzy-AHP. The proposed method is applied to determine the importance weights of the criteria for creating marketing mix elements of on-demand grocery delivery service in the fourth section. The fifth section provides the findings of the study. The last section presents conclusions, limitations, and future directions.

## Literature review

The literature is twofold: first, detailed explanations about the theoretical background of the study are presented. In the second part of the literature review, the studies employing the multi-criteria decision-making (MCDM) methods to evaluate online shopping are examined.

### Theoretical background

The fundamental elements used in the development of marketing strategies are known as the marketing mix. Although this term was first used by Borden [8], McCarthy gained the term more recognition by drawing attention to the fact that these essential elements are: price, product, place, and promotion, known as 4 Ps [25]. Then, Booms and Bitner [7] proposed a specific marketing mix of seven elements for services, arguing that people, processes, and physical evidence should be added to the four key elements. In many subsequent studies, it has been suggested that the marketing mix should change with the effect of technology and digitalization [35]. In addition, due to the personal use of digital resources, it has been argued that elements such as website design, customer service, and privacy can be added to the marketing mix [25]. In the study of Chen [11], it was suggested that the type of payment systems, personalization, and communication policy should be among the marketing mix in activities carried out via the internet channel. Extant literature has identified many factors that may be considered essential criteria for each marketing mix element of on-demand grocery delivery service. The main criteria encountered in the literature and determined by the experts are indicated in Table 1.

**Table 1:** The criteria for creating marketing mix for on-demand grocery delivery service

Marketing mix elements	Criteria	References	Definition
Product	Product quality (C_1_)	[37, 55, 62, 71]	A product’s brand equity and overall superiority over other alternatives [6]
	Wide range and categories (C_2_)	[2, 37, 55, 60, 62]	Breadth and depth of products offered to customers [48]
	The reputation of the store (C_3_)	[38, 37]	A multidimensional valuable asset that can arise from the factors such as quality, good customer service, and innovative products [69]
Price	Prices relative (C_4_)	[2, 20, 13, 37, 62]	Prices compared to prices of other online shopping platforms [13]
	Discount (C_5_)	[37, 61, 71]	Reduction in the standard selling price of a product
	Delivery costs (C_6_)	[61]	Amount of money paid for home delivery [61]
Promotion	Advertising (C_7_)	[61]	Means of communication with the customers using social networks advertising, mobile advertising, contextual advertising, native advertising, and display advertising [50]
	Public relations (C_8_)	[61]	All activities carried out to maintain a favorable public image using social media marketing, referral marketing, and content marketing [50]
	Sales promotion (C_9_)	[61]	Short-term incentive to attract customers using marketing communication activities such as e-mail marketing, social media call to action, and webinars [50]
Process	Order cancellation (C_10_)	[62]	The practice of making an order void after the order but before delivery [62]
	On-time delivery (C_11_)	[13, 72]	Delivery at the time promised [13]
	Order accuracy (C_12_)	[31]	It is the state that the products and items purchased have the promised features [31]
	Order tracking (C_13_)	[13]	Ability to track orders until delivery of the goods [13]
People	The attitude of customer service representative (C_14_)	[62, 37, 73]	Service-related attitudes and behaviors [21] of customer service representatives
	The attitude of courier (C_15_)	Expert opinion	Service-related attitudes and behaviors [21] of a courier
	Online ratings and review (C_16_)	[13, 73, 37]	Expressions of customer satisfaction or dissatisfaction [19]
Place	Working hours (C_17_)	[61]	Hours served to customers [61]
	Market supply (C_18_)	[61]	Number of residential areas served [61]
	The effectiveness of reverse logistics (C_19_)	Expert opinion	The effectiveness of returning the products received by the customers to the seller [27]
Physical evidence	Mobile store aesthetics (C_20_)	[37]	Aesthetic features of visual environments in which products are presented, explained, and promoted
	Professional appearance and manners of courier (C_21_)	Expert opinion	Attributes that appeal to consumers’ manners such as tone of voice and the appearance of couriers
	Quality and condition of equipment (C_22_)	Expert opinion	Appearance of equipment such as packaging and condition of vehicles used for home delivery

As seen in Table 1, this paper has focused on seven marketing mix elements. *Product* element is defined as the item presented to the market to meet a want or a need. It must be suitable for obtaining, using, consuming, or attracting customers [41]. In a study based on retail sale experiences, a model for the electronic marketing mix was created. It stated that the product element may refer to product variety and product assortment [35]. A study found that product variety significantly affects the perceptions and satisfaction of consumers who shop online [48]. Another study argued that most of the decisions made by online market shopping platforms regarding the product are related to which products will be kept in stock, how much, and what variety. It additionally revealed that the variety and availability of products positively affect consumers’ purchasing behavior [22]. Product quality is also considered one of the most critical criteria in online grocery shopping [76].

*Price* element is the only revenue-generating element in the marketing mix [41]. Pricing decisions in online sales are as important as traditional pricing decisions. In addition, there is greater price competition among the companies that sell online, so the importance of standardization of prices is critical [1]. Discounts and coupons are considered among pricing strategies. Using price promotions, the shopping platforms’ price images can be changed, and companies’ perceived value can be increased [22].

*Promotion* element demonstrates how a company is committed to communicating its products’ characteristics and persuading target customers to buy their products [41]. A study on online grocery shopping argued that the main criteria regarding promotion are advertising, sales promotion, and public relations [61].

*Place* element covers the mobile applications for online grocery shopping platforms [50]. The place also includes distribution channels [25]. One of the most significant features of the place element is maximizing the availability of sales channels [50]. When examining online grocery shopping platforms, the days and hours that the platform provides distribution services to customers can be evaluated within the place element [61].

*People* element was put forward by Judd [32], emphasizing that employees represent companies against customers. It was argued that unless employees are adequately trained on communicating with customers, any miscommunication may frustrate all marketing efforts [42]. This element is considered an essential element of the marketing mix because service consists of a performance, and performance cannot be separate from the performer [58].

*Process *element determines the method and order of services; it ensures that the value proposition promised to customers is created [56]. A poorly designed process can lead to a slow, useless, and low-quality service delivery resulting in customers’ frustration [42]. In some cases, after customers place an order, their orders may be canceled as companies are out of stock. Customers can give three types of reactions in this situation. They can accept buying substitute products, change the online shopping platform, or go off the internet [15]. A study reveals that factors such as late or incomplete online grocery delivery significantly affect customer satisfaction [14]. In some cases, failure to deliver quickly can cause consumers to abandon the online grocery shopping platform that they use [76].

*Physical evidence* element in e-commerce is divided into two components: traditional physical and virtual. While the physical environment is represented by delivery points, offline stores, and offices of the company; the virtual environment includes the website or mobile applications of online shopping platforms [50].

### The MCDM methods used to evaluate online shopping

Wen et al. [70] used data envelopment analysis to measure the relative effectiveness of e-commerce firms. Kong and Liu [40] employed the Fuzzy AHP method in their study to evaluate the success criteria in B2C e-commerce. In another study, Sun and Lin [66] applied the Fuzzy TOPSIS method to evaluate B2C e-commerce sites’ competitive advantage.

Kang et al. [36] examined B2C e-commerce sites’ service quality using the Fuzzy TOPSIS method. The study concluded that the method in question is a suitable method for evaluating the service quality of e-commerce sites. Chiu et al. [13] applied the DANP and VIKOR methods to examine the criteria that affect customer satisfaction in online shopping. They proposed a hybrid model for developing an online shopping business. Dey et al. [18] proposed an evaluation model for selecting online shopping sites using the Fuzzy AHP and Fuzzy TOPSIS methods. Chen et al. [12] examined the factors affecting customers’ online shopping decisions and the causal relationships between these factors employing the DEMATEL and ANP methods. Kahraman et al. [34] used the Hesitant Fuzzy Linguistic AHP method in their study to rank online shopping platforms. Rouyendegh et al. [53] used the AHP method and the Intuitionistic Fuzzy Technique together in their study to measure and evaluate the performance of e-commerce sites. Using the AHP and Intuitionistic Fuzzy TOPSIS methods, another study revealed that customers prefer online shopping platforms according to e-satisfaction indexes [3]. Kaushik et al. [37] applied the AHP and VIKOR methods to identify and rank the criteria for selecting online fashion retailers.

Li and Sun [43] combined Fuzzy AHP and TOPSIS-Grey Methodology to assess the success criteria for a B2C e-commerce website. Torkayesh et al. [67] proposed a hybrid BWM and WASPAS model to determine the importance weights of criteria for online retail shopping and assess digital suppliers. The literature indicates that the MCDM methods have been widely used to evaluate online shopping platforms’ performance, service quality, adoption, and determine the success criteria of these platforms, however, these methods have been used less frequently in online grocery shopping studies. Many of the previous studies employ one method or combined methods based on only expert opinions.

Different from the other studies, this paper applies the Buckley Extension-Based Fuzzy-AHP method to evaluate the criteria for creating the marketing mix (7Ps) of the on-demand grocery shopping service which has become increasingly popular, especially after the outbreak of COVID-19.

## Research methodology

This section provides the theoretical background of the Buckley Extension-Based Fuzzy-AHP method and summarizes how the method application is designed.

### Fuzzy sets

Fuzzy sets were introduced in 1965 by Zadeh [75]. Since then, the ordinary fuzzy sets were evolved by the following extensions: interval-valued fuzzy sets, type-2 fuzzy sets, intuitionistic fuzzy sets, fuzzy multisets, neutrosophic sets, nonstationary fuzzy sets, hesitant fuzzy sets, and Pythagorean fuzzy sets [33].

Due to the subjective perceptions and experiences of people involved in the decision-making processes, it is often not possible to make precise and fixed-value assessments on numerous real-life problems [68]. Instead, in such cases, it is more appropriate for decision makers to make evaluations with intermittent values that give safer results [24]. Fuzzy numbers (TFNs) consisting of three parts can be used against uncertainty in the fuzzy set theory. TFNs used in binary comparisons consist of three real numbers and a TFN can be indicated as (b, c, a). These numbers b, c, and a denote the smallest possible value, the most promising value, and the largest possible value, respectively. The membership function can be specified as follows:

1 $$ \mu \left( {x/\tilde{M}} \right) = \left\{ {\begin{array}{ll} {0,} & {x < b} \\ {\begin{array}{l} {\begin{array}{l} {\left( {x - b} \right)/\left( {c - b} \right),} \\ {\left( {a - x} \right)/\left( {a - c} \right),} \\ \end{array} } \\ {0,} \\ \end{array} } & {\begin{array}{l} {\begin{array}{l} {b \le x \le c} \\ {c \le x \le a} \\ \end{array} } \\ {x > a} \\ \end{array} } \\ \end{array} } \right. $$

Mathematical calculations including the addition, subtraction, multiplication, and arithmetic operations are defined in the following five equations, respectively, for the two triangle fuzzy numbers: $\tilde{B}_{1} = \left( {b_{1} ,c_{1} ,a_{1} } \right)$ and $\tilde{B}_{2} = \left( {b_{2} ,c_{2} ,a_{2} } \right)$

2 $$ \tilde{B}_{1} + \tilde{B}_{2} = \left( {b_{1} + b_{2} ,c_{1} + c_{2} ,a_{1} + a_{2} } \right) $$

3 $$ \tilde{B}_{1} - \tilde{B}_{2} = \left( {b_{1} - a_{2} ,c_{1} - c_{2} ,a_{1} - b_{2} } \right) $$

4 $$ \tilde{B}_{1} \otimes \tilde{B}_{2} = \left( {b_{1} \otimes b_{2} ,c_{1} \otimes c_{2} ,a_{1} \otimes a_{2} } \right) $$

5 $$ k \otimes \tilde{B}_{1} = \left( {k \otimes b_{1} ,k \otimes c_{1} ,k \otimes a_{1} } \right),\left( {k > 0} \right) $$

6 $$ \frac{{\tilde{A}_{1} }}{k} = \left( {\frac{{b_{1} }}{k},\frac{{c_{1} }}{k},\frac{{a_{1} }}{k}} \right),\left( {k > 0} \right) $$

TFNs used in this study and what these numbers mean are indicated in Table 2.

**Table 2:** Triangular fuzzy conversion scale

Linguistic scale	TFNs/reciprocal TFNs
AS—absolutely strong	(3.50, 4.00, 4.50)
VS—very strong	(2.50, 3.00, 3.50)
FS—fairly strong	(1.50, 2.00, 2.50)
SS—slightly strong	(0.50, 1.00, 1.50)
E—equal	(1.00, 1.00, 1.00)
SW—slightly weak	(0.67, 1.00, 2.00)
FW—fairly weak	(0.40, 0.50, 0.67)
VW—very weak	(0.29, 0.33, 0.40)
AW—absolutely weak	(0.22, 0.25, 0.29)

### Buckley extension-based fuzzy-AHP algorithm

**Fig. 1:**
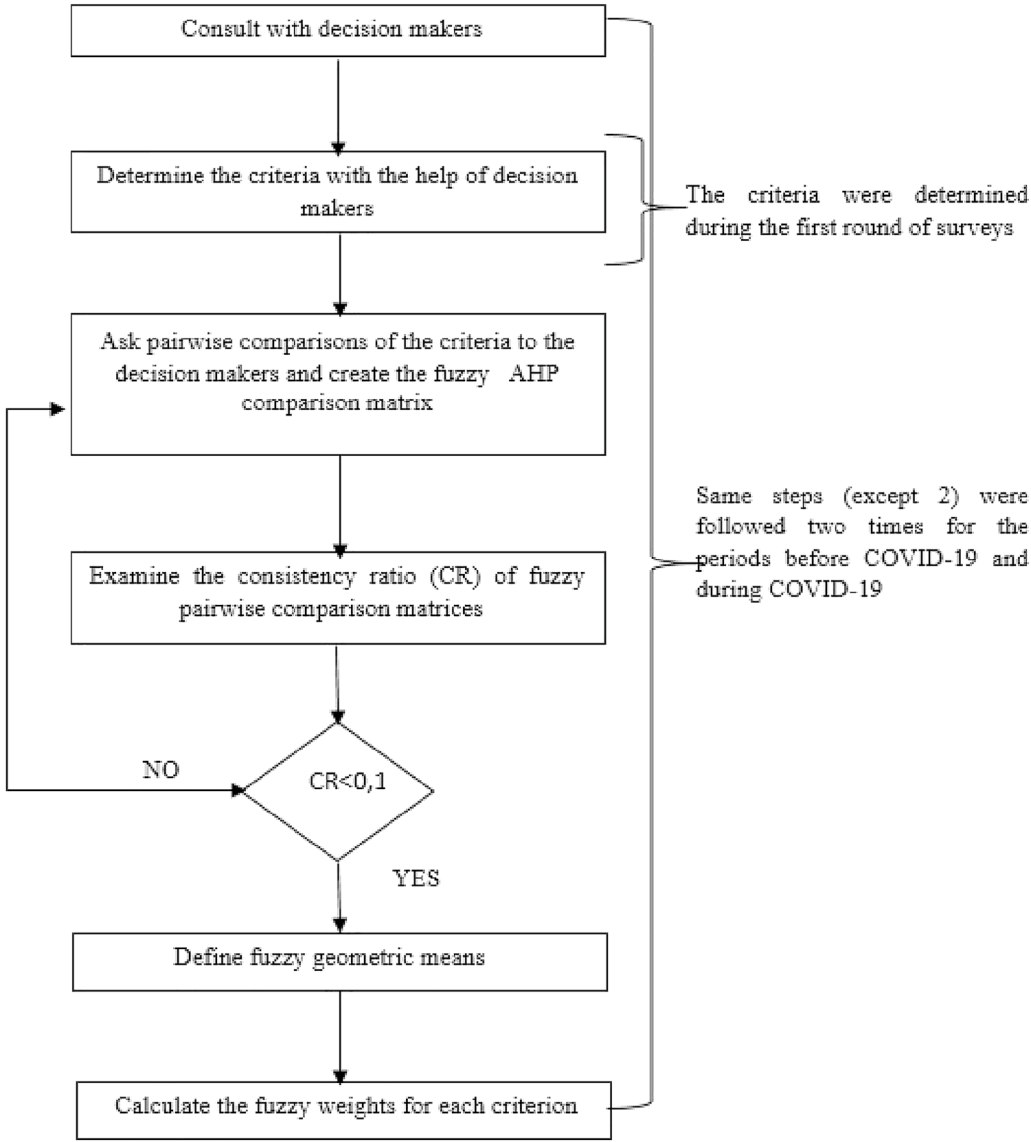
Flowchart of the main implementation steps of the method

Fuzzy AHP is a widely used multi-criteria decision-making method to solve hierarchical fuzzy problems [23]. Even though the analytical hierarchy process (AHP) proposed by Saaty in 1980 [54] offers a systematic and logical approach to solve planning and decision-making problems, it fails to offer solutions to some real-life problems that are not free of subjective perceptions and experiences of people [16]. Fuzzy AHP is a powerful method that can cope with the subjectivity of decision makers. The method provides linguistic variables that can authentically reflect the expert opinions, thereby ensuring the calculation of importance weights of criteria [17]. This paper employed the Buckley’s Fuzzy-AHP method to determine the weights of the criteria since it guarantees an original solution for the binary comparison matrix, and its steps can be shown more concisely than other Fuzzy approaches [24]. The main implementation steps of the method [9, 74] are illustrated in Fig. 1.

These steps are explained as follows [9]:

**Step 1:** Consult with decision makers.

**Step 2:** Determine the criteria with the help of decision makers.

**Step 3:** Create the fuzzy AHP binary comparison matrix.

At this step, the importance of each criteria is determined by comparing each criteria with one another using the TFNs. The equation explaining the creation of the matrix is as follows:

7 $$ \tilde{M} = \left\| {\begin{array}{llll} 1 & {\tilde{a}_{12} }
& {...} & {\tilde{a}_{1n} } \\ {\tilde{a}_{21} } & 1
& {...} & {\tilde{a}_{2n} } \\ \vdots & \vdots &
\ddots & \vdots \\ {\tilde{a}_{n1} } & {\tilde{a}_{n2} }
& {...} & 1 \\ \end{array} } \right\| = \left\|
{\begin{array}{llll} 1 & {\tilde{a}_{12} } & {...} &
{\tilde{a}_{1n} } \\ {1/\tilde{a}_{21} } & 1 & {...} &
{\tilde{a}_{2n} } \\ \vdots & \vdots & \ddots & \vdots
\\ {1/\tilde{a}_{n1} } & {\tilde{a}_{n2} } & {...} & 1
\\ \end{array} } \right\| $$

The values that the criteria can take in paired comparison are shown in the following equation:

8 $$ \tilde{a}_{ij} = \left\{ {\begin{array}{*{20}c} {\tilde{1},\tilde{3},\tilde{5},\tilde{7},\tilde{9}} \\ 1 \\ {\tilde{1}^{ - 1} ,\tilde{3}^{ - 1} ,\tilde{5}^{ - 1} ,\tilde{7}^{ - 1} ,\tilde{9}^{ - 1} } \\ \end{array} } \right. $$

In Eq. 8, if criterion i is more important than criterion j, it can take the first row values. If criterion i is as important as criterion j, it equals the value “1”. If it is less critical than criterion j, it can take the third row’s values.

**Step 4:** Examine the consistency of fuzzy pairwise comparison matrices.

**Step 5:** Define fuzzy geometric means using the geometric mean technique:

9 $$ \tilde{r}_{i} = \left( {\tilde{a}_{i1} \otimes \tilde{a}_{i2} \otimes ...\tilde{a}_{in} } \right)^{1/n} $$

The $\tilde{a}_{in}$ in Eq. 9 represents the fuzzy comparative value according to the n criterion of the i criterion. Therefore, the equation’s result corresponds to the geometric mean of the fuzzy comparative value of the i criterion according to the other criteria.

**Step 6:** Calculate the fuzzy weights for each criterion:

10 $$ \tilde{w}_{i} = \tilde{r}_{i} \otimes \left( {\tilde{r}_{1} \oplus \tilde{r}_{2} \oplus ...\tilde{r}_{n} } \right)^{ - 1} $$

$\tilde{w}_{i}$ in Eq. 10 corresponds to the fuzzy weight of each criterion. As stated before, triple fuzzy numbers can be used in the fuzzy set theory. Therefore, it is possible to show $\tilde{w}_{i}$ as “$\tilde{w}_{i} = \left( {bw_{i} ,cw_{i} ,aw_{i} } \right)$”.

## Application

The Buckley Extension-Based Fuzzy-AHP was applied for the purpose of determining the criterion weights for marketing mix elements of on-demand grocery shopping service based on expert opinion comparatively for the period before COVID-19 and during COVID-19. “The period before COVID-19” refers to the time before March 10th, which is the date of the first Covid case seen in Turkey. “During COVID-19” refers to the period after May 26th, which marks the last day of curfew in Turkey. In line with the purpose of the study, a survey was first conducted with five experts between 21 and 26 February when there were no recorded coronavirus cases in Turkey. At this step, a total of 22 criteria were determined and evaluated by the experts. The second round of surveys was conducted with the same experts between June 19 and 27 to determine their assessments for the time when the dramatic changes in consumers’ shopping behavior were seen. The experts include two professors in marketing, two senior executives, and a marketing professional.

Although all assessments on the criteria for the seven marketing mix elements are known, the fuzzy pairwise comparisons of criteria, fuzzy geometric means, and fuzzy weights are presented for only product element for the sake of brevity. The results of consistency measurements, Best Non-Fuzzy Performance (BNP) values, and rankings of the criteria are presented for all elements.

The fuzzy pairwise comparisons of the product criteria before COVID-19 and during COVID-19 are shown in Table 3.

**Table 3:** The pairwise comparisons of the product criteria

		Before COVID-19			During COVID-19		
		C_1_	C_2_	C_3_	C_1_	C_2_	C_3_
**C**_**1**_	Ex1	(1,1,1)	(1,1,1)	(0.5,1,1.5)	(1,1,1)	(0.5,1,1.5)	(0.67,1,2)
	Ex2	(1,1,1)	(1,1,1)	(0.67,1,2)	(1,1,1)	(1,1,1)	(0.67,1,2)
	Ex3	(1,1,1)	(1,1,1)	(1.5,2,2.5)	(1,1,1)	(0.5,1,1.5)	(0.5,1,1.5)
	Ex4	(1,1,1)	(0.5,1,1.5)	(1,1,1)	(1,1,1)	(0.5,1,1.5)	(0.4,0.5,0.67)
	Ex5	(1,1,1)	(0.67,1,2)	(0.5,1,1.5)	(1,1,1)	(1.5,2,2.5)	(0.5,1,1.5)
**C**_**2**_	Ex1	(1,1,1)	(1,1,1)	(0.5,1,1.5)	(1,1,1)	(1,1,1)	(0.4,0.5,0.67)
	Ex2	(1,1,1)	(1,1,1)	(0.67,1,2)	(1,1,1)	(1,1,1)	(0.67,1,2)
	Ex3	(1,1,1)	(1,1,1)	(1.5,2,2.5)	(1,1,1)	(1,1,1)	(1,1,1)
	Ex4	(1,1,1)	(1,1,1)	(0.67,1,2)	(1,1,1)	(1,1,1)	(0.29,0.33,0.40)
	Ex5	(1,1,1)	(1,1,1)	(1.5,2,2.5)	(1,1,1)	(1,1,1)	(0.67,1,2)
**C**_**3**_	Ex1	(0.67,1,2)	(0.67,1,2)	(1,1,1)	(0.5,1,1.5)	(1.5,2,2.5)	(1,1,1)
	Ex2	(0.5,1,1.5)	(0.5,1,1.5)	(1,1,1)	(0.5,1,1.5)	(0.5,1,1.5)	(1,1,1)
	Ex3	(0.4,0.5,0.67)	(0.4,0.5,0.67)	(1,1,1)	(0.67,1,2)	(1,1,1)	(1,1,1)
	Ex4	(1,1,1)	(0.5,1,1.5)	(1,1,1)	(1.5,2,2.5)	(2.5,3,3.5)	(1,1,1)
	Ex5	(0.67,1,2)	(0.4,0.5,0.67)	(1,1,1)	(0.67,1,2)	(0.5,1,1.5)	(1,1,1)

The consistency ratios of the fuzzy pairwise comparisons are shown in Tables 4 and 5.

**Table 4:** The consistency ratios before COVID-19

Marketing mix elements	Expert 1	Expert 2	Expert 3
Product	0.000	0.000	0.000
Price	0.033	0.033	0.000
Promotion	0.033	0.000	0.033
Process	0.064	0.064	0.016
People	0.033	0.033	0.033
Place	0.000	0.000	0.000
Physical evidence	0.000	0.000	0.033

Marketing mix elements	Expert 4	Expert 5	Aggregated
Product	0.000	0.033	0.006
Price	0.033	0.000	0.019
Promotion	0.033	0.000	0.019
Process	0.027	0.027	0.039
People	0.000	0.033	0.026
Place	0.033	0.033	0.013
Physical evidence	0.033	0.000	0.013

**Table 5:** The consistency ratios during COVID-19

Marketing mix elements	Expert 1	Expert 2	Expert 3
Product	0.000	0.000	0.000
Price	0.000	0.056	0.033
Promotion	0.033	0.000	0.000
Process	0.064	0,062	0.064
People	0.000	0.000	0.033
Place	0.000	0.033	0.033
Physical evidence	0.033	0.000	0.033

Marketing mix elements	Expert 4	Expert 5	Aggregated
Product	0.056	0.000	0.011
Price	0.056	0.000	0.019
Promotion	0.000	0.000	0.006
Process	0.064	0.064	0.063
People	0.056	0.000	0.017
Place	0.000	0.000	0.013
Physical evidence	0.033	0.000	0.019

The fuzzy geometric means and fuzzy weights of the product criteria are shown in Tables 6 and 7, respectively.

**Table 6:** The fuzzy geometric means of the product criteria

	Before COVID-19			During COVID-19		
C_1_	(1,1,1)	(1,1,0.8)	(1,0.8,1)	(1,1,1)	(0.42,0.49,0.59)	(0.9,1,1.11)
C_2_	(1,1,1)	(1,1,1)	(1,1,1)	(1,1,1)	(1,1,1)	(1.33,1.89,2.41)
C_3_	(0.62,0.87,1.32)	(0.87,1.32,0.48)	(1.32,0.48,0.76)	(0.9,1,1.11)	(0.41,0.53,0.75)	(1,1,1)

**Table 7:** The fuzzy weights of the product criteria

	Before COVID-19	During COVID-19
C_1_	(0.343,0.347,0.343)	(0.284,0.278,0.275)
C_2_	(0.386,0.364,0.345)	(0.433,0.436,0.426)
C_3_	(0.271,0.289,0.312)	(0.283,0.286,0.299)

The BNP values and rankings of criteria for all marketing mix elements are indicated in Table 8.

**Table 8:** The BNP values and rankings of criteria for marketing mix elements

		Before COVID-19		During COVID-19	
		BNP values	Rankings	BNP values	Rankings
Product	C_1_	0.344	2	0.279	3
	C_2_	0.365	1	0.432	1
	C_3_	0.290	3	0.289	2
Price	C_4_	0.369	1	0.388	1
	C_5_	0.343	2	0.388	2
	C_6_	0.288	3	0.224	3
Promotion	C_7_	0.370	2	0.339	1
	C_8_	0.387	1	0.329	3
	C_9_	0.243	3	0.333	2
Process	C_10_	0.376	1	0.258	2
	C_11_	0.279	2	0.416	1
	C_12_	0.169	4	0.186	3
	C_13_	0.177	3	0.140	4
People	C_14_	0.316	3	0.246	3
	C_15_	0.321	2	0.344	2
	C_16_	0.362	1	0.409	1
Place	C_17_	0.338	1	0.362	2
	C_18_	0.332	2	0.397	1
	C_19_	0.331	3	0.241	3
Physical evidence	C_20_	0.357	1	0.301	3
	C_21_	0.331	2	0.368	1
	C_22_	0.312	3	0.331	2

## Findings

**Fig. 2:**
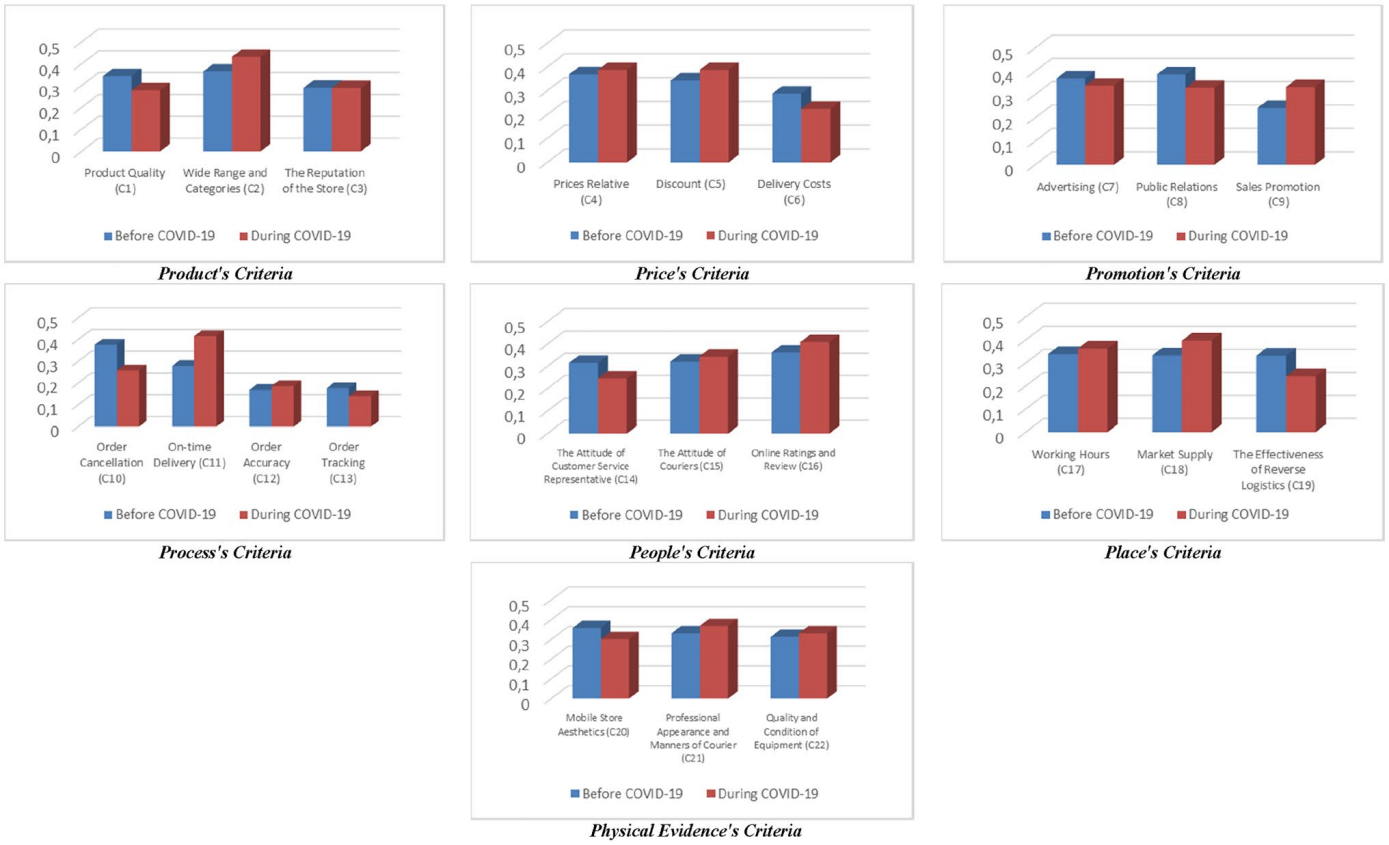
Changes in the Importance of all criteria

The importance weights of many criteria have significantly changed between the pre-COVID-19 and during COVID-19 periods. The changes are clearly shown in Fig. 2. According to the survey results, “wide range and categories” is the most crucial criterion among the criteria for the product element, with 36.5% in the period before COVID-19. This outcome supports the findings of Schulz et al. [57], who emphasize a broad assortment of products to be an indispensable criterion for successful online grocery retailing. This criterion is followed by “product quality” with 34.4%. The least essential criterion is “the reputation of the store” with 29%. “Wide range and categories” has been determined as the most critical criterion again, with 43.2% during COVID-19. This criterion is followed by “the reputation of the store” with 28.9%. The least essential criterion has been determined as “product quality” with 27.9%. Considering the criteria for the price element, “relative prices” is the most crucial criterion, with 36.9%. This criterion is followed by “discount” and “delivery costs” with 34.3% and 28.8%, respectively, before the pandemic. The findings show that “relative prices” and “discount” criteria are of equal importance, with 38.8%, while the importance weight of the “delivery costs” has decreased to 22.4% during the pandemic.

In light of the outcomes, the most crucial criterion among the criteria for the promotion element is “advertising” with 38.7% before the pandemic. “Advertising” is followed by “sales promotion” with 37%. The least essential criterion is “public relations” with 24.3%. On the other hand, the survey results show that the criteria’s importance weights are almost equal during the pandemic.

Considering the criteria for the process element, “on-time delivery” has been determined as the most important criterion, with 37.6% in the pre-pandemic period. The findings are in line with those of Otim and Grover [49], who underlined the role of on-time delivery for the customers’ repurchase intention. The second most significant criterion is “order accuracy” with 27.9%, while the importance weights of “order tracking” and “order cancellation” are below 20%. The findings support Changchit et al.'s [10] outcome of order accuracy as a strong determinant of online shopping behavior, and the results of Jalil [31] reported a significant impact of traceability of shipments in online shopping behavior. The survey results indicate that the most important criterion during the pandemic is “order accuracy” with 41.6%. “On-time delivery” criterion is the second at the ranking with 25.8%, while the importance weights of “order tracking” and “order cancellation” criteria have remained below 20%.

According to the outcomes, “online ratings and review” is the most crucial criterion among the criteria for the people element, with 36.2% in the period before the pandemic. This criterion is followed by “the attitude of courier” criterion with 32.1% and “the attitude of customer service representative” criterion with 31.6%, respectively. According to Limayem et al. [44], finer customer service is a significant determinant of shopping intention. The most important criterion is “online ratings and review” with 40.9% during the pandemic. The second most critical criterion is “the attitude of courier” with 34.4%. The least critical criterion is “the attitude of customer service representative” with 24.6%.

When the criteria for the place element are examined, it is seen that the importance weights of “working hours,” “market supply,” and “the effectiveness of reverse logistics” criteria are almost equal in the period before the pandemic. A study on online retailing indicates that ease of return positively affects repurchase intention [46]. “Market supply” has been determined as the most crucial criterion, with 39.7% during the pandemic. This criterion is followed by “working hours” with 36.2%, and “the effectiveness of reverse logistics” with 24.1%.

Considering the criteria for the physical evidence element, “mobile store aesthetics” has been determined as the most critical criterion in the period before the pandemic, with 35.7%. “Professional appearance and manners of courier” and “quality and conditions of equipment” have been found to have importance weights of 33.1% and 31.2%, respectively. However, the outcomes show that the most significant criterion is “professional appearance and manners of courier” with 36.8% during the pandemic. The second most critical criterion is “quality and conditions of equipment” with 33.1%, and the least essential criterion is mobile store aesthetics, with 30.1%.

## Conclusion

COVID-19 pandemic has led to significant changes in individuals’ grocery shopping habits and expectations. Despite the normalization process and the restrictions being lifted, many people continue to shop online for groceries. This study aims to determine the importance of the criteria for creating a marketing mix for on-demand grocery delivery service for the pre-COVID-19 and during COVID-19 periods. It is also aimed to determine which criteria have gained more importance during COVID-19 than in the period before COVID-19. In light of the study’s findings, it can be concluded that a “wide range and categories” criterion is the most important criterion for the product element for the two periods. Considering the price element, consumers have become more sensitive to “price-related criteria” except for delivery costs during COVID-19. “Public relations criterion” has stood out among the criteria for the promotion element and gained significant importance during COVID-19. Considering the process element, order accuracy has become much more critical during the COVID-19 pandemic. Two criteria for the place element (market supply and working hours) that maximize sales channels’ availability have gained importance during the COVID-19 pandemic. The visual criteria including “the professional appearance and manners of courier” as well as “quality and conditions of equipment” have also gained importance during the COVID-19 pandemic.

The original contributions of this study are as follows: (1) it applies an intelligent comparative approach to determine the importance weights of the criteria for marketing mix elements of the on-demand grocery delivery service; (2) it reveals the criteria that have gained importance during the COVID-19 pandemic; (3) it provides clues for the decision makers in times of similar pandemics and crises that are likely to be seen in the future. This paper has some limitations that may pave the way for further studies. First, in this study, five experts were surveyed due to time constraints caused by the uncertainty of the arrival date of the COVID-19 to Turkey after the first case was reported in China. If an accurate timing in data collection via enough number of experts was not followed, it would not be possible to evaluate the criteria comparatively for the period before COVID-19 and during COVID-19. Future studies may include more experts. Another limitation is with regard to the nature of the pandemic. The unstable spreading trend of the pandemic may hinder the predictability of consumer behaviors. As a future suggestion, it is possible to analyze and rank the on-demand grocery delivery companies’ marketing performance using combined MCDM methods. In addition, the causal relationships between the criteria can be analyzed using experimental designs.
